# Prevalence of, association with, severity of, and prognostic role of serum hemoglobin level in acutely decompensated heart failure patients

**DOI:** 10.1186/s12872-023-03510-6

**Published:** 2023-10-04

**Authors:** Sepehr Omoomi, Maryam Heidarpour, Najmeh Rabanipour, Mona Saadati, Omid Vakilbashi, Davood Shafie

**Affiliations:** 1 Cardiology/Heart Failure and Transplantation, Heart Failure Research Center, Isfahan Cardiovascular Research Institute, Isfahan University of Medical Sciences, Isfahan, Iran; 2 Endocrinology, Isfahan Endocrine and Metabolism Research Center, Isfahan University of Medical Sciences, Isfahan, Iran; 3 Department of Biostatistics and Epidemiology, School of Public Health, Isfahan University of Medical Science, Isfahan, Iran

**Keywords:** Heart failure, Anemia, Polycythemia, Mortality

## Abstract

**Background:**

The role of hemoglobin (Hb) level in the short-term prognosis of patients with acute decompensated heart failure (ADHF) remains a matter of debate. We aimed to declare the prevalence of, association with, severity of, and prognostic role of SHL with ADHF.

**Methods:**

Using the data from the Persian Registry Of Cardiovascular Disease/ Heart Failure (PROVE-HF) study, we assessed the association between anemia and polycythemia (Hb < 13 g/dLit, > 16.5 g/dLit in males and < 12 g/dLit, and > 16 g/dLit in females, respectively) and short-term mortality using Cox proportional hazard modeling, with adjustment of clinically relevant variables.

**Results:**

Of 3652 ADHF patients, anemia was seen in 1673 patients (48.40%). The prevalence of mild, moderate, and severe anemia was 42.33% (n = 1546), 3.23% (n = 118), and 0.24% (n = 9), respectively. Also, 422 patients (11.55%) had polycythemia. Compared to non-anemic patients, anemic patients were mainly male, older, and were more likely to have diabetes mellitus (DM), renal dysfunction, hypertension (HTN), and thyroid disease. Significant predictors of short-term mortality were lower systolic and diastolic blood pressure, lower Hb level, and higher blood urea nitrogen (BUN). Anemic patients had higher all-cause mortality [adjusted hazard ratio (aHR) 1.213, 95% confidence interval [CI] 1.054–1.396]. Moderate anemia increased mortality by approximately 80% in males (aHR 1.793, 95% CI 1.308–2.458) and females (aHR 1.790, 95% CI 1.312–2.442), respectively. Polycythemia had no association with short-term mortality in both genders (P-value > 0.05).

**Conclusions:**

This study revealed that anemia is an adverse prognostic factor for short-term mortality in ADHF patients, with higher mortality in moderately anemic patients.

**Supplementary Information:**

The online version contains supplementary material available at 10.1186/s12872-023-03510-6.

## Introduction

Anemia is of considerable importance because of being a common comorbid disease in patients suffering from heart failure (HF) regardless of their left ventricular ejection fraction (LVEF), one of the most common types of cardiovascular disease in the world [1–6].

The prevalence of anemia in HF patients ranges from 5 to 70% based on the demographic characteristics of the studied population, differences in the definitions of anemia, and lack of information about correctable causes of anemia [7, 8].

The inception of anemia in HF patients is associated with multifactorial reasons, including iron metabolism deficits, inflammation, chronic diseases, bone marrow suppression, chronic kidney disease (CKD), hemodilution, and some guideline-recommended medication to treat HF patients [9–13].

Theoretically, anemia induces an increase in heart workload through an increase in preload, a decrease in peripheral vascular resistance (PVR), and an increase in cardiac output (CO), leading to worsening HF [8]. HF causes renal failure because of the reduced output and may ultimately induce anemia by decreasing the secretion of endogenous erythropoietin leading to a vicious cycle called cardio-renal-anemia syndrome [14, 15].

Despite the interaction between anemia and HF, there are controversies regarding the role of anemia being an independent risk factor for worsening HF and a mediator of poor prognosis or a marker of increased risk [16]. There is limited confounder-adjusted evidence regarding both the short- and long-term impact of anemia on the prognosis of HF patients [17, 18].

Our study aimed to investigate the effect of admission Hb level and 1-year mortality in acute decompensated heart failure (ADHF) patients. Additional objectives were to define the risk factors associated with being anemic in ADHF patients and adjust them based on the demographic and clinical features of the population.

## Method

### Study design

We performed this study in the context of the Persian Registry Of Cardiovascular Disease/ Heart Failure (PROVE/HF) study [19]. Briefly, it is a registry of HF patients’ data based on the international classification of disease, 10th revision (ICD-10) from 18 distinct cardiac centers in Isfahan province, Iran, launched in March 2015. The data were gathered continuously through a questionnaire containing 27 parts comprising demographic, underlying, and comorbid diseases leading to HF, past medical history, pre-admission medication usage, and any medical treatment implemented during hospitalization from medical records of hospital archives by trained data collectors. This study was conducted under the supervision of the Isfahan University of Medical Sciences (IUMS), and the ethical committee of IUMS approved this study (IR.ARI.MUI.REC.1401.153).

### Study participants

It was a single-center retrospective study from March 2016 to March 2020, which reviewed the clinical data of all HF patients over 18 in the Isfahan Province, Iran. Patients with underlying malignant diseases, primary bone marrow diseases, concurrent corticosteroid-based medication, active bleeding, hereditary hemoglobinopathies like thalassemia and sickle-cell anemia, hemolytic anemia, megaloblastic anemia, anemia due to nutrient deficiencies, and those who died during hospitalization or were without follow-up since discharge were excluded.

### Data collection and definition

Data on demographic features of the studied population, smoking status, pre-admission and discharge medication [beta-blockers (BBs), angiotensin-converting enzyme inhibitors (ACEIs), angiotensin receptor blockers (ARBs), diuretics, mineralocorticoid receptor antagonists (MRAs), digoxin, and nitrates], and length of hospital stay (LOS) were extracted from medical records of patients. We gathered comorbid diseases, including diabetes mellitus (DM), ischemic heart disease (IHD), hypertension (HTN), stroke, chronic obstructive pulmonary disorders (COPD), CKD, and thyroid diseases. We also extracted the laboratory data, including sodium (mEq/Lit), potassium (mEq/Lit), hemoglobin (Hb) (g/dLit) on admission, blood urea nitrogen (BUN) (mg/dLit), and creatinine on admission and discharge. Serum creatinine level was used to calculate the estimated glomerular filtration rate (eGFR) using the chronic kidney disease epidemiology collaboration (CKD-EPI) equation and define the renal functional capacity (RFC); subsequently. According to World Health Organization (WHO) criteria, anemia and polycythemia were defined as Hb concentration of < 13 g/dLit, > 16.5 g/dLit in males and < 12 g/dLit, and > 16 g/dLit in females, respectively. Among the anemic patients, mild, moderate, and severe anemia was defined as Hb ≥ 9.1 g/dLit, 6.1 g/dLit ≤ Hb < 9 g/dLit, and Hb < 6 g/dLit, respectively [12, 20]. Body mass index (BMI) was calculated by division of weight (Kg) over the square of height (m^2^). We collected systolic blood pressure (SBP), diastolic blood pressure (DBP), and left ventricle ejection fraction (LVEF) on admission. Further stratification of anemia-, RFC-, age-, LVEF-, and LOS- status is presented in Table 1. The primary endpoint was all-cause mortality in 1-year. (Table 1)

**Table 1:** Baseline, clinical, and laboratory characteristics of the study population based on hemoglobin status in the total population

Variables		Total (n = 3652)	Anemia		P
			Yes (n = 1346)	No (n = 2306)	
Age(years) < 65		1139 (31.2)	387 (28.8)	752 (32.6)	0.015
Males (%)		2281 (62.5)	876 (65.1)	1405 (60.9)	0.012
BMI (kg/m2)		26.44± 3.72	26.21± 3.85	26.57± 3.63	0.005
Ischemic heart disease (%)		3016 (82.6)	1110 (82.5)	1906 (82.7)	0.885
Diabetes mellitus (%)		1728 (47.3)	714 (53)	1014 (44)	< 0.001
Hypertension (%)		2418 (66.2)	927 (68.9)	1491 (64.7)	0.009
Kidney diseases (%)		971 (26.6)	488 (36.3)	483 (20.9)	< 0.001
Thyroid disorders (%)		269 (7.4)	131 (9.7)	138 (6)	< 0.001
Smoking status (%)		612 (16.8)	205 (15.2)	407 (17.6)	0.059
Systolic blood pressure (mmHg)		131.09± 27.34	129.18± 27.83	132.20± 27.63	0.002
Diastolic blood pressure (mmHg)		81.32± 16.09	79.29± 15.70	82.50± 16.20	< 0.001
Sodium (mEq/l)		138.85± 4.83	138.28± 4.80	139.18± 4.82	< 0.001
Potassium (mEq/l)		4.49± 0.64	4.56± 0.69	4.45± 0.60	< 0.001
Blood urea nitrogen (mg/dl)		28.15± 14.87	32.10± 17.19	25.85± 12.79	< 0.001
CKD Stage based on eGFR on Admission	Normal or High	115 (3.1)	76 (5.6)	39 (1.7)	< 0.001
	Mildly Decreased	478 (13.1)	265 (19.7)	213 (9.2)	
	Mildly to Moderately Decreased	989 (27.1)	380 (28.2)	609 (26.4)	
	Moderately to Severely Decreased	1022 (28)	329 (24.4)	693 (30.1)	
	Severely Decreased	901 (24.7)	257 (19.1)	644 (27.9)	
	Kidney Failure	147 (4)	39 (2.9)	108 (4.7)	
CKD Stage based on eGFR at Discharge	Normal or High	95 (2.6)	70 (5.2)	25 (1.1)	< 0.001
	Mildly Decreased	816 (22.3)	348 (25.9)	468 (20.3)	
	Mildly to Moderately Decreased	1772 (48.5)	619 (46)	1153 (50)	
	Moderately to Severely Decreased	608 (16.6)	198 (14.7)	410 (17.8)	
	Severely Decreased	316 (8.7)	95 (7.1)	221 (9.6)	
	Kidney Failure	45 (1.2)	16 (1.2)	29 (1.3)	
EF groups	< 30	2159 (59.1)	732 (20)	1427 (39.1)	< 0.001
	30–39	743 (20.3)	308 (22.9)	435 (18.9)	
	40–49	368 (10.1)	146 (10.8)	222 (9.6)	
Discharge drug history	Beta-blockers (%)	2905 (79.5)	1083 (80.5)	1822 (79)	0.295
	ACEIs/ARBs (%)	3026 (82.9)	1121 (83.3)	1905 (82.6)	0.603
	mineralocorticoid receptor antagonists (%)	1449 (39.7)	469 (34.8)	980 (42.5)	< 0.001
	Diuretics (%)	2372 (65)	903 (67.1)	1469 (63.7)	0.039
Follow-up death (%)		866(23.7)	383(28.5)	483(20.9)	< 0.001

BMI: body mass index, EF: ejection fraction, ACEIs: angiotensin-converting enzyme inhibitors, ARBs: angiotensin receptor blockers, CKD: chronic kidney disease

* Results from independent t-test and chi-square test

### Statistical analysis

Continuous variables were reported by mean ± standard deviation (SD) or median (interquartile range). Distinct variables were presented by number and percentage. Before additional analysis, Shapiro-Wilk and Leven’s tests were performed to determine the normality and homogeneity of data. Patients were categorized based on their gender-, age-, LVEF-, RFC-, and admission Hb- status. Based on the data homogeneity, an Independent T-test/ analysis of variance (ANOVA) with post hoc test or Mann-Whitney-U test and Chi-square or Fisher’s exact tests were performed to compare continuous and distinct variables, respectively, among anemic and non-anemic individuals according to their baseline features. Kaplan-Meier curve and log-rank test were performed for the primary endpoint. Univariate and multivariate logistic regression models were implemented to assess the factors correlated with anemia. Factors with a P-value < 0.05 in univariate analysis were entered in multivariate analysis. Adjustment covariables in the multivariable analysis included age, BMI, IHD, DM, HTN, CKD, thyroid disorders, smoking status, creatinine on admission, SBP, DBP, sodium, potassium, BUN, RFC both on admission and at discharge, EF groups and discharged drug consumption (BBs, ACE-Is, ARBs, MRAs, nitrates, and diuretics). The Odds ratio (OR) and 95% confidence interval (95% CI) were reported. Both unadjusted and adjusted Cox proportional hazard regression models were performed to estimate the impact of admission Hb value on the primary endpoint. Statistical Package for Social Sciences (SPSS Inc., version 22.0, Chicago, IL, USA) was used to perform all analyses.

## Result

### Clinical characteristics of the study population

During the study period, we included all the 3896 eligible patients in the registry. Among them, 244 patients were excluded due to a lack of baseline data and mortality during admission. Regarding the WHO criteria, anemic individuals, including 1546 (42.33%), 118 (3.23%), and 9 (0.24%) with mild, moderate, and severe anemia, respectively, formed 45.8% of the enrolled population. The mean age was 70.01 ± 12.64 years, and 62.5% were male.

The baseline clinical, laboratory characteristics, and clinical endpoint of enrolled individuals are presented in Table 1. The independent predictors of anemia at hospital admission included male gender, older age, DM, HTN, CKD, thyroid diseases, and higher potassium and LVEF levels. Supplementary Tables 1 and 2 present the comparison of clinical and laboratory characteristics and clinical endpoint of individuals based on their Hb status in males and females, respectively. (Supplementary Tables 1 and 2) Kidney and thyroid diseases were observed significantly more in moderately anemic individuals than in polycythemic individuals. Further, moderately anemic ones had higher Na, K, and BUN levels and lower DBP than polycythemic ones. However, the non-anemic group had a more severe CKD status on admission and discharge. Mildly and moderately anemic individuals were less likely to have LVEF < 30% compared to non-anemic patients. Also, they received fewer mineralocorticoid receptor antagonists and more diuretics. There was a reverse association between age and Hb concentration in males.

### Survival analysis and primary outcome

**Fig. 1:**
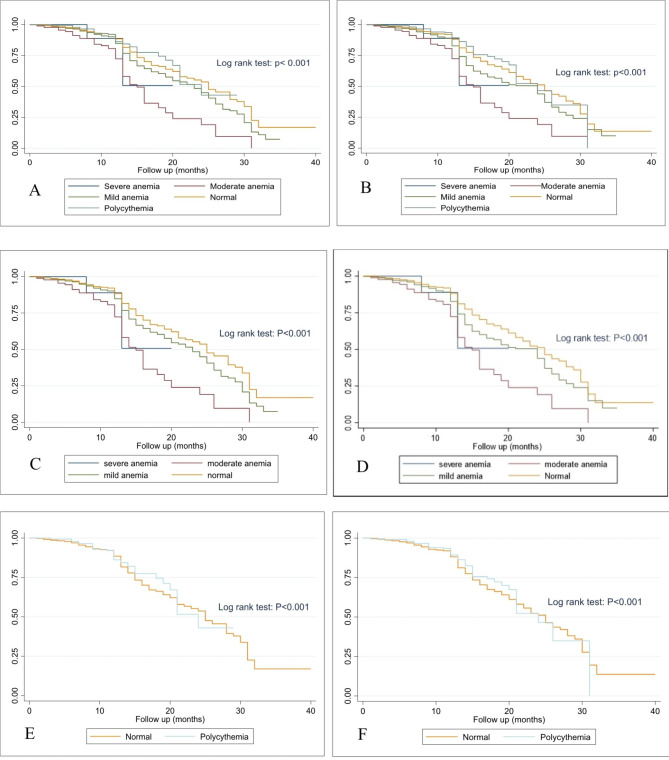
Kaplan-Meier survival curve of the studied population. (**A**) all-cause mortality in 40 months of male patients. (**B**) all-cause mortality in 40 months of female patients. (**C**) all-cause mortality in 40 months of male patients with severe, moderate, and mild anemia and normal Hb. (**D**) all-cause mortality in 40 months of female patients with severe, moderate, and mild anemia and normal Hb. (**E**) all-cause mortality in 40 months of male patients with polycythemia and normal Hb. (**F**) all-cause mortality in 40 months of female patients with polycythemia and normal Hb

The mean duration of follow-up was 10.95 ± 7.31 months. During the study period, 866 individuals (23.71%), including 28.5% and 20.9% of anemic and non-anemic individuals, respectively, died (P-value < 0.001). Table 2 represents the univariate and multivariate analyses of predictors of 1-year mortality for HF. Anemia was found to be an independent predictor of 1-year mortality for HF (Odds Ratio [OR] 1.31; 95% confidence interval [95% CI] 1.11–1.54; P-value: 0.002). Meanwhile, having a history of IHD and high SBP were found to be independent predictors of 1-year mortality (P-value < 0.001). Significant higher mortality was found in mildly and moderately anemic individuals compared to non-anemic ones regardless of their genders (P-value < 0.001). In addition, the mortality rate was significantly higher in moderately anemic compared to mildly anemic patients in both genders (43.2% vs. 25.7%, P-value < 0.001, and 43.2% vs. 27.4%, P-value < 0.001 in males and females, respectively). Kaplan Meier survival curves (Fig. 1) represent the significant survival difference between the anemic, polycythemic, and normal hemoglobin individuals with ADHF during the follow-up period (Log-Rank Test; P-value < 0.001) in both genders. Based on these curves, patients with normal Hb had higher survival compared to anemic and polycythemic patients in both genders. Meanwhile, severely and mildly anemic patients had the lowest and highest survival rate among anemic ones in both genders. Further, mild anemic patients had higher survival compared to polycythemic patients (Log-Rank Test; P-value < 0.001).

**Table 2:** Univariate and multivariate analyses of predictors of 1-year heart failure mortality during the follow-up period

Variables		Univariate analysis		Multivariate analysis	
		OR (95% CI)	P	OR (95% CI)	P
Anemia		1.50 (1.28–1.75)	< 0.001	1.31 (1.11–1.54)	0.002
Age(years) < 65		1.869 (1.562–2.237)	< 0.001	1.851 (1.520–2.255)	< 0.001
IHD (%)		1.302 (1.074–1.580)	0.007	1.339 (1.097–1.635)	0.004
SBP (mmHg)		1.008 (1.005–1.011)	< 0.001	1.010 (1.006–1.014)	< 0.001
Potassium (mEq/l)		0.686 (0.610–0.772)	< 0.001	0.791 (0.698–0.896)	< 0.001
BUN (mg/dl)		0.981 (0.976–0.985)	< 0.001	0.992 (0.986–0.999)	0.022
Discharge drug history	ACEIs/ARBs (%)	1.390 (1.146–1.685)	0.001	1.343 (1.101–1.640)	0.004

IHD: ischemic heart disease, SBP: systolic blood pressure, BUN: blood urea nitrogen, ACEIs: angiotensin converting-enzyme inhibitors, ARBs: angiotensin receptor blockers, CKD: chronic kidney disease

Besides anemia, this table depicts only variables found to be predictive (or with a trend to be predictive) of the primary endpoint in the multivariate model. Other variables predictive of the primary endpoint in univariate analyses, including the history of kidney diseases, sodium level, DBP, and CKD stage on admission, entered the multivariate model. However, they were not independent predictors of 1-year mortality for heart failure

In Cox regression models, anemia was a strong predictor of 1-year mortality. (Table 3) The crude and adjusted Cox regression hazard ratio of follow-up death stratified by Hb status (Table 4) revealed that moderate anemia is independently associated with 1-year mortality in both genders (Adjusted Hazard Ratio [HR] 1.793; 95% CI 1.308–2.458; P-value < 0.001 and adjusted HR 1.790; 95% CI 1.312–2.442; P-value < 0.001 in male and female, respectively). In addition, mild anemia is independently associated with 1-year mortality in the female gender (adjusted HR 1.208; 95% CI 1.031–1.416; P-value = 0.019). However, although mild anemia is associated with 1-year mortality in the male gender, it is not an independent factor to impact mortality (Crude [HR] 1.277; 95% CI 1.108–1.470; P-value = 0.001 vs. Adjusted [HR] 1.139; 95% CI 0.984–1.318; P-value = 0.081).

**Table 3:** Univariate and multivariate cox regression hazard ratio of the primary endpoint during the follow-up period

Variables	Univariate analysis		Multivariate analysis	
	HR (95% CI)	P	HR (95% CI)	P
Anemia	1.440 (1.258–1.647)	< 0.001	1.213 (1.054–1.396)	0.007
Age(years) < 65	0.558 (0.474–0.656)	< 0.001	0.587 (0.492–0.699)	< 0.001
SBP (mmHg)	0.993(0.991–0.996)	< 0.001	0.992 (0.989–0.996)	< 0.001
DBP (mmHg)	0.994 (0.989–0.998)	0.005	1.006 (1.000 -1.011)	0.048
Sodium (mEq/l)	0.970 (0.959-982)	< 0.001	0.977 (0.964–0.989)	< 0.001
Potassium (mEq/l)	1.306 (1.184–1.440)	< 0.001	1.119 (1.010–1.240)	0.031
BUN (mg/dl)	1.019 (1. 015-1.023)	< 0.001	1.010 (1.005–1.016)	< 0.001

SBP: systolic blood pressure, BUN: blood urea nitrogen

Besides anemia, this table depicts only variables found to be predictive (or with a trend to be predictive) of the primary endpoint in the multivariate model. Other variables predictive of the primary endpoint in univariate analyses, including the history of kidney diseases, ACEIs/ARBs medication at discharge, and CKD stage on admission, entered the multivariate model. However, they were not independent predictors of 1-year mortality for heart failure

**Table 4:** Crude and adjusted Cox regression hazard ratio of mortality follow-up based on the Hb level

Variables	Model	Hb level								
		Severe Anemia	P	Moderate Anemia	P	Mild Anemia	P	Normal	Polycythemia	P
Follow-up death in male (%)	Crude	2.107 (0.786–5.646)	0.138	2.494 (1.861–3.341)	< 0.001	1.277 (1.108–1.470)	0.001	1	0.899 (0.629–1.283)	0.556
	Adjusted*	1.727 (0.637–4.681)	0.283	1.793 (1.308–2.458)	< 0.001	1.139 (0.984–1.318)	0.081	1	0.883 (0.616–1.266)	0.499
Follow-up death in female (%)	Crude	2.055 (0.768-5.50)	0.152	2.429 (1.821–3.242)	< 0.001	1.374 (1.182–1.597)	< 0.001	1	0.862 (0.643–1.156)	0.322
	Adjusted*	1.730 (0.639–4.682)	0.281	1.790 (1.312–2.442)	< 0.001	1.208 (1.031–1.416)	0.019	1	0.825 (0.612–1.111)	0.206

*Adjusted for age, body mass index, ischemic heart disease, diabetes mellitus, hypertension, kidney diseases, thyroid disorders, smoking status, systolic blood pressure, diastolic blood pressure, sodium, potassium, blood urea nitrogen, chronic kidney disease (CKD) stage both on admission and at discharge, left ventricle ejection fraction (EF) groups and discharged drug consumption (beta-blockers, angiotensin-converting enzyme inhibitors, angiotensin receptor blockers, mineralocorticoid receptor antagonists and diuretics)

## Discussion

In this study, anemia was found to be an independent risk factor for 1-year mortality. The prevalence of anemia in our study was approximately 36.85%, similar to that observed in the meta-analysis conducted by Groenveld, the Polish cohort, and the Swedish HF registry [16, 21, 22]. However, a lower prevalence of anemia was reported in the Italian Network on Congestive Heart Failure (IN-CHF) registry and the Valsartan Heart Failure (Val-HeFT) trial due to the younger population, outpatient nature of participants, and their ethnicities [6, 23]. A recent study conducted in Italy reported a higher prevalence of anemia among the whole population (45%) and ADHF patients (59%) compared to us. This could be due to the short study period, older participants, and smaller sample size in comparison to larger studies [24].

We discovered that older age, male gender, DM, and CKD were independent risk factors of anemia on admission. Further, anemic patients had higher cardiac and non-cardiac comorbidities. Although both the anemic and non-anemic patients were overweight, the anemic patients had lower BMI, which may be explained by their older age.

Several mechanisms are contributed to the onset and aggravation of anemia, including comorbid kidney diseases, blunted erythropoietin synthesis/response, hemodilution, cytokine-mediated inflammation, some guideline-recommended medications, and disturbances in iron metabolism [9–13, 25]. Meanwhile, iron deficiency anemia, blunted erythropoietin synthesis/response, and guideline-recommended treatments are assumed to be the most significant underlying factors responsible for the inception and exacerbation of anemia in HF patients [26].

The Efficacy of Vasopressin Antagonism in Heart Failure Outcome Study with Tolvaptan (EVEREST) study and the IN-CHF Registry demonstrated that anemic patients received less guideline-recommended medication, including BBs and ACEI/ARBs [6, 27]. Also, several mechanisms are considered for ACEI/ARBs, and some BBs, including carvedilol, inhibit erythropoiesis [28–30]. However, recent studies reported that sacubitril/valsartan (Neprilysin/Angiotensin Receptor Inhibitor) reduces anemia in patients with CRS [31, 32]. MRAs may induce erythropoiesis by down-regulating hepcidin. It controls the body’s iron metabolism by ferroportin-1 and leads to the impairment of duodenal iron absorption and the release of internal iron storage from reticuloendothelial cells [33]. The result of our study demonstrates that anemic patients received fewer MRAs and ACEI/ARBs (RAAS inhibitors) than non-anemic patients. The possible explanation is anemic ones received fewer RAAS inhibitors due to the anemia-induced vasodilation that leads to lower blood pressure (Supplementary Table 1). However, among the non-anemic patients, polycythemic ones received fewer RAAS inhibitors. Meanwhile, the observed difference was not significant.

A prevalence of anemia in heart failure with preserved EF (HFpEF) patients was reported higher than in heart failure with reduced EF (HfrEF) patients with no comparable differences in their renal functional capacity (RFC). Caughey MC et al. declared that anemic patients with HFpEF received fewer diuretics and supported the possibility of hemodilution-induced anemia in HFpEF patients [34]. However, HFpEF is not participated in our observed outcome due to being an exclusion criterion.

Pathogenic pathways of CKD-induced anemia include relative erythropoietin deficiency, aggregation of uremic-induced erythropoiesis inhibitors, shortened RBC survival, and impaired iron metabolism by hepcidin as a fundamental mechanism of CKD-induced anemia [32, 33]. Anemic patients had higher BUN serum levels. However, our study revealed better RFC in anemic patients than non-anemic patients on admission and discharge. A significantly aggressive diuretic treatment therapy possibly explained this in anemic patients compared to non-anemic patients. Aggressive diuretic therapy leads to less venous congestion, better arterial perfusion as well as higher natriuresis and diuresis, and subsequent lower effective intravascular volume. Lower SBP, DBP, sodium levels, and higher BUN levels in anemic patients support this hypothesis.

The result of our study indicates that anemia is not only the marker of worse prognosis but also an independent risk factor of short-term mortality in ADHF patients. It is independently associated with a 21.3% increase in 1-year mortality. The independently higher impact of anemia on mortality was not observed in severely anemic patients of both genders and is simply the marker of a worse prognosis. A relatively low number of severely anemic patients, a higher burden of comorbidities, and a higher possibility of mortality rate during hospitalization in severely anemic patients can explain it. Moderate anemia is independently associated with an approximately 80% increase in the mortality risk in both genders. In this regard, the EVEREST, IN-CHF registry, Val-HeFT, and previous smaller studies reported that anemia independently increases the risk of mortality and morbidity in HF patients [6, 23, 27]. A recent systematic review and meta-analysis confirm that anemia is an independent prognostic factor of mortality in HF patients [35]. Meanwhile, it did not define the impact of severity and gender difference on the prognostic effect of anemia on mortality. However, the Italian and Polish studies found no impact of anemia on HF prognosis. The Polish study found mild-to-moderate anemia as a marker of older age, worse clinical conditions, and a higher comorbidity burden. The Italian study revealed the lack of impact of anemia on HF prognosis by co-analyzing the congestion parameters. The controversial results of these studies can be explained by the exclusion of severely anemic patients, older participants, and a lower sample size [16, 24].

Our study revealed that mild anemia is only a marker of worse prognosis in males and is independently associated with a higher 20.8% mortality risk in females. A possible explanation can be the gender-related differences in cardiovascular diseases. Previous literature revealed that females formed a higher proportion of patients with HFpEF because of valvular and hypertensive etiologies [36]. The Framingham study reported a higher impact of hypertension in developing HF in females than males (three-fold vs. two-fold increase, respectively) [37–39]. Females are more susceptible to the deleterious effects of greater pulsatile and early arterial load on diastolic function and ventricular-arterial interaction [40, 41]. Despite the lack of HFpEF patients in our study, females formed a higher proportion of higher EF than males, which could explain this. Further, mild anemic patients had higher BP than non-anemic patients. It was defined that the higher the Hb level, the lower the impact of Hb on mortality observed in non-anemic patients. However, the observed association was not significant.

Our study has several limitations. First, due to the observational nature of research and the lack of information about the etiology of anemia, identifying the underlying mechanisms of the inception of anemia was impossible. Similarly, we had no data to differentiate primary and secondary polycythemia. Some previous studies reported the different thrombotic risks and mortality in ADHF patients. Second, we have no data on Hb at discharge to differentiate transient from persistent anemia. Third, most of our patients were HF-exacerbated patients; Thus, we could not compare the new-onset ones with exacerbated ones. Some previous studies reported the lack of influence of anemia on mortality in new-onset HF. Fourth, due to the recent prevalent use of sodium-glucose cotransporter-2 (SGLT2) inhibitors in the recommended treatment and their potency to counteract plasma volume expansion and further possible additional benefits for anemic patients is of interest. However, we could not evaluate the impact of these drugs in this study due to the lack of SGLT2 inhibitors during the implementation of the study, especially at the beginning, in our country. Finally, some patients with any severity of anemia who died in the hospital were excluded and may influence the analysis.

## Conclusion

This study demonstrated confirmatory evidence regarding the significant prognostic influence of moderate anemia on short-term mortality and the possibility of being the target in treating ADHF patients. Further, we provided evidence regarding the lack of mild and severe anemia on short-term mortality. We also provided evidence regarding the independent influence of mild anemia on the short-term mortality of female patients.

### Electronic supplementary material

Below is the link to the electronic supplementary material.

Supplementary Material 1

## Data Availability

The datasets generated during and/or analyzed during the current study are not publicly available due to confidential issues but are available from the corresponding author at reasonable request.

## List of abbreviations

Hb: Hemoglobin
SHL: Serum hemoglobin level
HF: Heart failure
CVD: Cardiovascular disease
ADHF: Acute decompensated heart failure
PVR: Peripheral vascular resistance
PROVE/HF: Persian Registry Of cardioVascular diseasE/Heart Failure
IUMS: Isfahan University of Medical Sciences
DM: Diabetes mellitus
HTN: Hypertension
COPD: Chronic obstructive pulmonary disease
BMI: Body mass index
ACEIs: Angiotensin-converting enzyme inhibitors
ARBs: Angiotensin receptor blockers
BB: Beta-blocker
MRA: Mineralocorticoid receptor antagonists
SBP: Systolic blood pressure
DBP: Diastolic blood pressure
IHD: Ischemic heart disease
CKD: Chronic kidney disease
eGFR: estimated glomerular filtration rate
WHO: World health organization
LVEF: Left ventricular ejection fraction
BUN: Blood urea nitrogen
Cr: Creatinine
SD: Standard deviation
ANOVA: Analysis of variance
aHR: adjusted Hazard ratio
OR: Odds ratio
SPSS: Statistical Package for Social Sciences
RAAS: Renin-Angiotensin-Aldostron-System
