# Supplementary material for: Prevalence of, association with, severity of, and prognostic role of serum hemoglobin level in acutely decompensated heart failure patients
Source: BMC Cardiovasc Disord. 2023 Oct 4;23:491. doi: 10.1186/s12872-023-03510-6 (PMC10552373; doi:10.1186/s12872-023-03510-6)
Supplement: Supplementary file 1 — Supplementary Material 1 [file 12872_2023_3510_MOESM1_ESM.docx]

Supplementary Table 1. Baseline, clinical, and laboratory characteristics of the study population based on hemoglobin status in males

| Variables | | Total  (n=3652) | Hb level | | | | | P |
| --- | --- | --- | --- | --- | --- | --- | --- | --- |
|  |  |  | Severe Anemia  (n=9) | Moderate Anemia  (n=118) | Mild Anemia  (n=1546) | Normal  (n=1806) | Polycythemia  Hb (n=173) |  |
| Age (years)  <65 | | 1139  (31.2) | 2  (22.2) | 32  (27.1) | 427  (27.6) ^a^ | 619  (34.3) | 59  (34.1) | 0.001 |
| Males (%) | | 2281  (62.5) | 4  (44.4) | 69  (58.5) | 846  (54.7) ^a^ | 1209  (66.9) | 153  (88.4) | < 0.001 |
| BMI (kg/m2) | | 26.44±  3.72 | 25.13±  4.59 | 26.36±  4.03 | 26.33±  3.87 | 26.53±  3.58 | 26.53±  3.47 | 0.455 |
| Ischemic heart disease (%) | | 3016  (82.6) | 7  (77.8) | 87  (73.7) | 1298  (84) ^e^ | 1486  (82.3) | 138  (79.8) | 0.046 |
| Diabetes mellitus (%) | | 1728  (47.3) | 6  (66.7) | 69  (58.5) | 812  (52.5) ^a^ | 764  (42.3) ^d^ | 77  (44.5) | < 0.001 |
| Hypertension (%) | | 2418  (66.2) | 8  (88.9) | 75  (63.6) | 1085  (70.2) ^a^ | 1144  (63.3) | 106  (61.3) | < 0.001 |
| Kidney diseases (%) | | 971  (26.6) | 5  (55.6) | 70  (59.3) ^b^ | 428  (31.2) ^a,e^ | 375  (20.8) ^d^ | 39  (22.5) | < 0.001 |
| Thyroid disorders (%) | | 269  (7.4) | 1  (11.1) | 26  (22) ^b^ | 140  (9.1) ^a,e^ | 96  (5.3) ^d^ | 6  (3.5) | < 0.001 |
| Smoking status (%) | | 612  (16.8) | 0 | 16  (13.6) ^b^ | 203  (13.1) ^a^ | 344  (19) ^j^ | 49  (28.3) ^c^ | < 0.001 |
| Systolic blood pressure (mmHg) | | 131.09±  27.34 | 127±  37.04 | 125.71±  28.80 | 130.85±  27.76 | 131.57± 27.59 | 132.03±  27.70 | 0.240 |
| Diastolic blood pressure (mmHg) | | 81.32±  16.09 | 74.22±  30.29 | 75.40±  16.53^b,e^ | 80.69±  15.83 | 82.09±  16.03 ^d^ | 83.18±  16.71 | < 0.001 |
| Sodium (mEq/l) | | 138.85±  4.83 | 137.10±  4.69 | 137.56±  4.62 ^b^ | 138.69±  4.91 | 139.02±  4.71 ^d^ | 139.50±  5.28 | 0.002 |
| Potassium (mEq/l) | | 4.49±  0.64 | 4.42±  0.98 | 4.63±  0.85 | 4.52±  0.66 ^a^ | 4.45±  0.60 ^d^ | 4.53±  0.64 | 0.001 |
| Blood urea nitrogen (mg/dl) | | 28.15±  14.87 | 29.79±  10.62 | 44.06±  22.95 ^b,e^ | 29.94±  15.54 ^a,f^ | 25.73±  12.85 ^d^ | 26.50±  12.72 ^c^ | < 0.001 |
| CKD Stage based on eGFR on Admission | Normal or High | 115  (3.1) | 1  (11.1) | 25  (21.2) ^b,e^ | 63  (4.1) ^a^ | 21  (1.2) ^d^ | 5  (2.9) | < 0.001 |
|  | Mildly Decreased | 478  (13.1) | 2  (22.2) | 35  (29.7) ^b,e^ | 273  (17.7) ^a^ | 157  (8.7) ^d^ | 11  (6.4) ^c^ |  |
|  | Mildly to Moderately Decreased | 989  (27.1) | 3  (33.3) | 34  (28.8) | 448  (29) | 462  (25.6) | 42  (24.3) |  |
|  | Moderately to Severely Decreased | 1022  (28) | 1  (11.1) | 13  (11) ^b,e^ | 415  (26.8) | 537  (29.7) ^d^ | 56  (32.4) |  |
|  | Severely Decreased | 901  (24.7) | 1  (11.1) | 8  (6.8) ^b,e^ | 305  (19.7) ^a^ | 539  (29.8) ^d^ | 48  (27.7) |  |
|  | Kidney Failure | 147  (4) | 1  (11.1) | 3  (2.5) | 42  (2.7) ^a^ | 90  (5) | 11  (6.4) |  |
| CKD Stage based on eGFR at Discharge | Normal or High | 95  (2.6) | 1  (11.1) | 19  (16.1) | 60  (3.9) ^a^ | 15  (0.8) ^d,h^ | 0 | < 0.001 |
|  | Mildly Decreased | 816  (22.3) | 3  (33.3) | 31  (26.3) ^b^ | 430  (27.8) ^a^ | 334  (18.5) | 18  (10.4) ^c^ |  |
|  | Mildly to Moderately Decreased | 1772  (48.5) | 4  (44.4) | 48  (40.7) ^b^ | 711  (46) | 909  (50.3) | 100  (57.8) ^c^ |  |
|  | Moderately to Severely Decreased | 608  (16.6) | 0 | 12  (10.2) | 230  (14.9) ^a^ | 333  (18.4) | 33  (19.1) |  |
|  | Severely Decreased | 316  (8.7) | 0 | 7  (5.9) | 101  (6.5) ^a^ | 189  (10.5) | 19  (11) |  |
|  | Kidney Failure | 45  (1.2) | 1  (11.1) | 1  (0.8) | 14  (0.9) | 26  (1.5) ^h^ | 3  (1.7) |  |
| EF groups | <30 | 2159  (59.1) | 4  (44.4) | 40  (33.9) ^b,e^ | 861  (55.7) ^a^ | 1153  (63.8) ^d^ | 101  (58.4) | < 0.001 |
|  | 30-39 | 743  (20.3) | 3  (33.3) | 44  (37.3) ^e^ | 340  (22) ^a^ | 316  (17.5) ^d^ | 40  (23.1) |  |
|  | 40-49 | 368  (10.1) | 0 | 14  (11.9) | 169  (10.9) | 171  (9.5) | 14  (8.1) |  |
| Discharge drug history | Beta-blockers (%) | 2905  (79.5) | 9  (100) | 91  (77.1) | 1234  (79.8) | 1439  (79.7) | 132  (76.3) | 0.413 |
|  | ACEIs/ARBs (%) | 3026  (82.9) | 7  (77.8) | 94  (79.7) | 1280  (82.8) | 1505  (83.3) | 140  (80.9) | 0.780 |
|  | mineralocorticoid receptor antagonists (%) | 1449  (39.7) | 3  (33.3) | 27  (22.9) ^e^ | 563  (36.4) ^a^ | 793  (43.9) ^d^ | 63  (36.4) | < 0.001 |
|  | Diuretics (%) | 2372  (65) | 7  (77.8) | 73  (61.9) | 1024  (66.2) | 1178  (65.2) | 90  (52) ^c,j^ | 0.005 |
| Follow-up death (%) |  | 866  (23.7) | 4  (44.4) | 51  (43.2) ^b,e^ | 398  (25.7) ^a^ | 380  (21) ^d^ | 33  (19.1) | < 0.001 |

BMI: body mass index, EF: ejection fraction, ACEIs: angiotensin-converting enzyme inhibitors, ARBs: angiotensin receptor blockers, CKD: chronic kidney disease

* Results from independent ANOVA and chi-square test.

a: P-values < 0.05 resulted from a comparison of Mild Anemia vs. Normal.

b: P-values < 0.05 resulted from a comparison of Moderate Anemia vs. Polycythemia.

c: P-values < 0.05 resulted from a comparison of Mild Anemia vs. Polycythemia.

j: P-values < 0.05 resulted from a comparison of Normal vs. Polycythemia.

d: P-values < 0.05 resulted from a comparison of Moderate vs. Normal.

e: P-values < 0.05 resulted from a comparison of Moderate Anemia vs. Mild Anemia.

f: P-values < 0.05 resulted from a comparison of Moderate Anemia vs. Severe Anemia.

g: P-values < 0.05 resulted from a comparison of Mild Anemia vs. Severe Anemia.

h: P-values < 0.05 resulted from a comparison of Normal vs. Severe Anemia.

i: P-values < 0.05 resulted from a comparison of Polycythemia vs. Severe Anemia.

Supplementary Table 2. Baseline, clinical, and laboratory characteristics of the study population based on hemoglobin status in females

| Variables | | Total  (n=3652) | Hb level | | | | | P |
| --- | --- | --- | --- | --- | --- | --- | --- | --- |
|  |  |  | Severe Anemia  (n=9) | Moderate Anemia  (n=118) | Mild Anemia  (n=950) | Normal  (n=2326) | Polycythemia  (n=249) |  |
| Age(years)  <65 | | 1139  (31.2) | 2  (22.2) | 32  (27.1) | 269  (28.3) | 753  (32.4) | 83  (33.3) | 0.138 |
| Female (%) | | 1371  (37.5) | 5  (55.6) | 49  (41.5) ^b^ | 447  (47.1) ^a^ | 830  (35.7) | 40  (16.1) ^c,j,i^ | < 0.001 |
| BMI (kg/m2) | | 26.44±  3.72 | 25.13±  4.59 | 26.36±  4.03 | 26.17±  3.66 | 26.53±  3.73 | 26.61±  3.58 | 0.085 |
| Ischemic heart disease (%) | | 3016  (82.6) | 7  (77.8) | 87  (73.7) | 788  (82.9) | 19.29  (82.9) | 205  (82.3) | 0.143 |
| Diabetes mellitus (%) | | 1728  (47.3) | 6  (66.7) | 69  (58.5) ^e^ | 511  (53.8) | 1024  (44) ^d^ | 118  (47.4) | < 0.001 |
| Hypertension (%) | | 2418  (66.2) | 8  (88.9) | 75  (63.6) | 676  (71.2) ^a^ | 1495  (64.3) | 164  (65.9) | 0.002 |
| Kidney diseases (%) | | 971  (26.6) | 5  (55.6) | 70  (59.3) ^b,e^ | 330  (34.7) ^a^ | 512  (22) ^d^ | 54  (21.7) ^c^ | < 0.001 |
| Thyroid disorders (%) | | 269  (7.4) | 1  (11.1) | 26  (22) ^b,e^ | 99  (10.4) ^a^ | 130  (5.6) ^d^ | 13  (5.2) | < 0.001 |
| Smoking status (%) | | 612  (16.8) | 0 | 16  (13.6) ^b^ | 118  (12.4) ^a^ | 409  (17.6) | 69  (27.7) ^c,j^ | < 0.001 |
| Systolic blood pressure (mmHg) | | 131.09±  27.34 | 127±  37.04 | 125.71±  28.80 | 130.85±  27.76 | 131.57± 27.59 | 132.03±  27.70 | 0.240 |
| Diastolic blood pressure (mmHg) | | 81.32±  16.09 | 74.22±  30.29 | 75.40±  16.53^b,e^ | 80.69±  15.83 | 82.09±  16.03 ^d^ | 83.18±  16.71 | < 0.001 |
| Sodium (mEq/l) | | 138.85±  4.83 | 137.10±  4.69 | 137.56±  4.62 ^b^ | 138.47±  4.10 ^a^ | 139.02±  4.70 ^d^ | 139.36±  5.31 | < 0.001 |
| Potassium (mEq/l) | | 4.49±  0.64 | 4.42±  0.98 | 4.63±  0.85 ^d^ | 4.55±  0.69 ^a^ | 4.45±  0.59 | 4.50±  0.63 | < 0.001 |
| Blood urea nitrogen (mg/dl) | | 28.15±  14.87 | 29.79±  10.62 | 44.06±  22.95 ^b,e^ | 31.73±  16.70 ^a,f^ | 26.03±  12.67 ^d^ | 26.76±  13.41 ^c^ | < 0.001 |
| CKD Stage based on eGFR on Admission | Normal or High | 115  (3.1) | 1  (11.1) | 25  (21.2) ^b,e^ | 52  (5.5) ^a^ | 32  (1.4) ^d^ | 5  (2) | < 0.001 |
|  | Mildly Decreased | 478  (13.1) | 2  (22.2) | 35  (29.7) ^b^ | 193  (20.3) ^a^ | 226  (9.7) ^d^ | 22  (8.8) ^c^ |  |
|  | Mildly to Moderately Decreased | 989  (27.1) | 3  (33.3) | 34  (28.8) | 276  (29.1) | 618  (26.6) | 58  (23.3) |  |
|  | Moderately to Severely Decreased | 1022  (28) | 1  (11.1) | 13  (11) ^b,e^ | 234  (24.6) ^a^ | 700  (30.1) ^d^ | 74  (29.7) |  |
|  | Severely Decreased | 901  (24.7) | 1  (11.1) | 8  (6.8) ^b,e^ | 174  (18.3) ^a^ | 642  (27.6) ^d^ | 76  (30.5) ^c^ |  |
|  | Kidney Failure | 147  (4) | 1  (11.1) | 3  (2.5) | 21  (2.2) ^a^ | 108  (4.6) | 14  (5.6) ^c^ |  |
| CKD Stage based on eGFR at Discharge | Normal or High | 95  (2.6) | 1  (11.1) | 19  (16.1) ^e^ | 47  (4.9) ^a^ | 28  (1.2) ^d,h^ | 0 | < 0.001 |
|  | Mildly Decreased | 816  (22.3) | 3  (33.3) | 31  (26.3) ^b^ | 291  (30.6) ^a^ | 458  (19.7) | 33  (13.3) ^c^ |  |
|  | Mildly to Moderately Decreased | 1772  (48.5) | 4  (44.4) | 48  (40.7) | 415  (43.7) ^a^ | 1171  (50.3) | 134  (53.8) ^j^ |  |
|  | Moderately to Severely Decreased | 608  (16.6) | 0 | 12  (10.2) | 129  (13.6) ^a^ | 418  (18) | 49  (19.7) |  |
|  | Severely Decreased | 316  (8.7) | 0 | 7  (5.9) | 59  (6.2) ^a^ | 220  (9.5) | 30  (12) ^c^ |  |
|  | Kidney Failure | 45  (1.2) | 1  (11.1) | 1  (0.8) | 9  (0.9) ^g^ | 31  (1.3) | 3  (1.2) |  |
| EF groups | <30 | 2159  (59.1) | 4  (44.4) | 40  (33.9) ^b,e^ | 515  (54.2) ^a^ | 1451  (62.4) ^d^ | 149  (59.8) | < 0.001 |
|  | 30-39 | 743  (20.3) | 3  (33.3) | 44  (37.3) ^b,e^ | 211  (22.2) | 433  (18.6) ^d^ | 52  (20.9) |  |
|  | 40-49 | 368  (10.1) | 0 | 14  (11.9) | 105  (11.1) | 232  (10) | 17  (6.8) |  |
| Discharge drug history | Beta-blockers (%) | 2905  (79.5) | 9  (100) | 91  (77.1) | 772  (81.3) | 1842  (79.2) | 191  (76.7) | 0.209 |
|  | ACEIs/ARBs (%) | 3026  (82.9) | 7  (77.8) | 94  (79.7) | 791  (83.3) | 1932  (83.1) | 202  (81.1) | 0.788 |
|  | mineralocorticoid receptor antagonists (%) | 1449  (39.7) | 3  (33.3) | 27  (22.9) ^b^ | 338  (35.6) ^a^ | 984  (42.3) ^d^ | 97  (39) | < 0.001 |
|  | Diuretics (%) | 2372  (65) | 7  (77.8) | 73  (61.9) | 644  (67.8) | 1511  (65) | 137  (55) ^c,j^ | 0.004 |
| Follow-up death (%) |  | 866  (23.7) | 4  (44.4) | 51  (43.2) ^b,e^ | 260  (27.4) ^a^ | 502  (21.6) ^d^ | 49  (19.7) | < 0.001 |

BMI: body mass index, EF: ejection fraction, ACEIs: angiotensin-converting enzyme inhibitors, ARBs: angiotensin receptor blockers, CKD: chronic kidney disease

* Results from independent ANOVA and chi-square test.

a: P-values < 0.05 resulted from a comparison of Mild Anemia vs. Normal.

b: P-values < 0.05 resulted from a comparison of Moderate Anemia vs. Polycythemia.

c: P-values < 0.05 resulted from a comparison of Mild Anemia vs. Polycythemia.

j: P-values < 0.05 resulted from a comparison of Normal vs. Polycythemia.

d: P-values < 0.05 resulted from a comparison of Moderate vs. Normal.

e: P-values < 0.05 resulted from a comparison of Moderate Anemia vs. Mild Anemia.

f: P-values < 0.05 resulted from a comparison of Moderate Anemia vs. Severe Anemia.

g: P-values < 0.05 resulted from a comparison of Mild Anemia vs. Severe Anemia.

h: P-values < 0.05 resulted from a comparison of Normal vs. Severe Anemia.

i: P-values < 0.05 resulted from a comparison of Polycythemia vs. Severe Anemia.
